# Prevalence and Factors of Addictive Internet Use among Adolescents in Wuhan, China: Interactions of Parental Relationship with Age and Hyperactivity-Impulsivity

**DOI:** 10.1371/journal.pone.0061782

**Published:** 2013-04-15

**Authors:** Xianhua Wu, Xinguang Chen, Juan Han, Heng Meng, Jianghong Luo, Liesl Nydegger, Hanrong Wu

**Affiliations:** 1 Department of Child and Adolescent Health and Maternal and Child Health, Huazhong University of Science and Technology, Wuhan, Hubei, China; 2 Pediatric Prevention Research Center, Wayne State University, Detroit, Michigan, United States of America; 3 Claremont Graduate University School of Community and Global Health, Claremont, California, United States of America; Catholic University of Sacred Heart of Rome, Italy

## Abstract

**Purposes:**

This study examined the prevalence of addictive Internet use and analyzed the role of parental relationship in affecting this behavior among a random sample of adolescents in Wuhan, China.

**Methods:**

Students (n = 1,101) were randomly selected from four schools, including 638 boys and 463 girls with a mean age of 13.8 (standard deviation = 1.2) years. Addictive Internet use, parental relationship, hyperactivity-impulsivity were measured by validated instruments. Prevalence rate, ANOVA and multiple linear regression method were used to analyze the level of Internet addiction and its association with parental relationship, hyperactivity-impulsivity, as well as the interaction of parental relationship with chronological age and hyperactivity-impulsivity.

**Results:**

The prevalence rate of Internet addiction was 13.5% (16.5% for boys and 9.5% for girls, *p*<0.01). Compared to non-addictive users, addictive Internet users were scored significantly lower on parental relationships and significantly higher on hyperactivity-impulsivity. Interaction analysis indicated that better parental relationship was associated with more reductions in risk of addictive Internet use for younger students than for older students, and with more risk of Internet addiction among higher than among lower hyperactivity-impulsivity students.

**Conclusions:**

Findings of this study indicate that adolescent addictive Internet use is a significant public health threat in China. Prevention interventions targeting parental relationship must consider adolescent’s age and hyperactivity-impulsivity tendency.

## Introduction

**An Explosive Growth in Netizens and Addictive Internet Use**

The number of people who use Internet or *netizens* in China has experienced an explosive growth in the past two decades. Survey data as well as technical records from China Internet Network Information Center (CNNIC) indicate that the total number of netizens in China increased from 0.62 million in 1997 to 126 million in 2006, and to 513 million by December, 2011 [1], [2]. Of these netizens, more than a half (56.5%) or approximately 300 million are young Chinese less than 30 years of age. These young netizens spend on average 18.7 hours per week online [2]. The large number of young Chinese netizens and the high intensity of Internet use indicate the urgency to examine addictive Internet use, particularly Internet addiction among young people and to investigate modifiable influential factors for effective prevention intervention.

Internet addiction, also known as pathological or addictive Internet use, was first recognized as a health issue in the United States in the mid-1990s [3], [4]. Dr. Goldberg, a New York psychiatrist, was believed to be the first person to propose the term *Internet addiction disorder* based on the criteria from the Diagnostic and Statistical Manual of Mental Disorder–IV (DSM-IV) [5]. Among a number of definitions for Internet addiction, the work by Young is of particular significance. She defined Internet addiction as an impulse-control disorder that does not involve an intoxicant similar to symptoms of pathological gambling. To facilitate Internet addiction research, she developed a measure, Young’s Internet Addiction Test [6], which has been widely used in reported research studies [7], [8].

### High Prevalence of Addictive Internet Use in China

Data from several studies indicate that the prevalence of Internet addition among the mainland Chinese varied from 8–10% with more males than females being addictive Internet users [9]–[12]. The rates are similar to those reported for youth in Hong Kong, Taiwan [13], and Korea [14]. Converging evidence from diverse sources indicates that adolescents who compulsively use the Internet are at increased risk to suffer from a number of negative social, behavioral, and health consequences, including depression, anxiety, loneliness [15]–[19], poor school performance, disorganized daily life and poor personal relationships [15], [18]–[22]. One study reported a strong association between addictive Internet use and drug use among Chinese adolescents [9]. It is of great public health significance to protect adolescents from addictive Internet use in order to avoid these negative health consequences.

### Poor Parental Relationship as a Risk Factor

To better understand Internet use and to more effectively protect adolescent from problematic use of Internet, researchers have found a number of risk factors, including intra-personal (e.g., age, gender), psychosocial (e.g., depression, anxiety, self-identity), and familial factors [21]–[23]. Among many of these factors, parental relationship may be of fundamental significance [14], [24], [25]. Research findings from different sources suggest that poor parental relationship may have played a key role in determining whether an adolescent who used the Internet would progress to an addictive Internet user [26]–[29]. Lack of parental monitoring [29]–[33], more parent-child conflict [26]–[28], [34], and feelings of parental rejection and punishment [26], [27], [35] are frequently associated with increased risk for addictive Internet use. Lack of parental caring and monitoring may channel adolescents to the Internet for emotional and social support [26], [36]; while adolescents with psychological problems due to prolonged Internet use may, in turn, go to the Internet for assistance, forming a vicious cycle [37].

### Age and Hyperactivity-impulsivity Modifying Parental Impact

Adolescence represents a period of rapid and unevenly paced physical and mental development [12]. Impact of parental relationship on development differs for adolescents at different ages [38], [39]. The strength of connection between parents and children decreases with age [40], [41]. Consequently, the impact of parental relationship on addictive Internet use may differ for adolescents at different ages. Developmentally, more parental support is needed for younger adolescents than for older adolescents to grow and develop [42]. Meanwhile, in addition to parental impact, a positive association between addictive Internet use and chronological age has been observed among adolescents [33], [43]. Furthermore, studies on other unhealthy behaviors revealed that the protective effects of parental monitoring on delinquent and problem behaviors are stronger for younger adolescents than for older adolescents [44]–[49]. These research findings imply that the impact of parental relationship on addictive Internet use in adolescents could also be modified by adolescents’ age. We hypothesize that the risk of poor parental relationship on addictive Internet use is greater for younger adolescents than for older adolescents. Understanding the interplay of parental relationship with adolescent’s age is of great significance for prevention of Internet addiction; however, no reported study has examined this interaction among Chinese youth.

In addition to adolescents’ age, levels of hyperactivity-impulsivity may modify the effect of parental relationship on addictive Internet use. As a subtype of Attention Deficit Hyperactivity Disorder (ADHD), hyperactivity-impulsivity is an established risk factor for many risky behaviors including addictive Internet use among adolescents [7], [43], [50]–[55]. Hyperactive and impulsive children are easy to notice but hard to ignore. They often experience high levels of negativity in parent-child interactions [56]–[58]. Mothers of hyperactive children were generally more negative during play and less responsive to child-initiated interactions [58], while adolescents who are hyperactive and impulsive often experience difficulties with parents [59], [60]. Consequently, youth with hyperactivity-impulsivity may be at increased risk to access and persistently use Internet, leading to heightened risk of addictive use. Therefore, it is very likely that the impact of parental relationship on addictive Internet use may differ for youth with different levels of hyperactivity-Impulsivity. Understanding this mechanism is of significance for targeted intervention, but no reported study has examined this issue among Chinese youth.

### Purposes of this Study

The present study sought to assess the prevalence rates of addictive Internet use among Chinese adolescents, to investigate the association of parental relationship with Internet addiction, and to evaluate the role of chronological age and hyperactivity-impulsivity in modifying the association between parental relationship and addictive Internet use. Findings of this study will advance our understanding of addictive Internet use and provide new data supporting behavioral interventions to protect children from addictive Internet use.

## Methods

### Study Design and Participants

Parental written informed consent and students’ written informed assent were obtained from all participants before they completed the survey. The study protocol was reviewed and proved by the Ethnic Committee of Tongji Medical College, Huazhong University of Science and Technology. Data used for this analysis was collected in 2009. Participants were recruited from public middle schools in Wuhan, a provincial capital city located in central China with a population of more than eight million in 2009 [61], [62]. Students were randomly selected employing a stratified cluster random sampling method in two steps. The first step was to select four typical middle schools with the following criteria: average school size, located in different geographic regions of Wuhan, representing different levels of quality of education, and willingness to participate. The second step was to randomly select students by class, and students in the selected classes on the survey day were then invited to participate. All students (*n* = 1,344) in 28 sampled classes from the four selected schools were invited, and 1,299 agreed to participate (response rate = 96.7%). Among these participants, 1,200 provided useful data, of whom 1,101 (91.7%) reported having access to the Internet.

Seven graduate students from Tongji Medical College were trained to conduct the survey. Data collection was completed in classroom settings. The trained data collectors distributed the survey questionnaires to the participating students and instructed them to complete the survey anonymously. Teachers, school administrators and staff were asked to leave while students were completing the questionnaire. The questionnaire consisted of four parts (1) demographic information, (2) Quality of Life Scale for Children and Adolescents [63], (3) Strengths and Difficulties Questionnaire (SDQ, Kid version) [64], and (4) Internet use. It took approximately 20–25 minutes for most students to complete the survey.

### Measures

#### Internet Addiction

Young’s Internet Addiction Test, Chinese version (YIAT-C) was used to assess Internet addiction. YIAT-C was derived from its English version for Chinese Internet users [8]. As in the original scale, it consisted of 20 items assessing the frequencies of 20 different types of Internet abuse behaviors (1 = “not at all” and 5 = “always”). A typical example is “How often do you try to cut down the amount of time you spend online but failed?” The 20 YIAT-C questions were embedded in the survey questionnaire for data collection. Data from reported studies indicate adequate reliability of the YIAT in languages other than English (Cronbach alpha ≥0.90) [65], [66]. A psychometric assessment of our data indicated that Cronbach alpha varied from 0.90 to 0.91 for different subgroups by gender, grade and age. Internet addiction was assessed by summing the YIAT-C scores (range 20 to 100, *M* = 36) and scores ≥50 were classified as Internet addicted [65].

#### Parental Relationship

Parental relationship was assessed using the Parental Relationship Subscale of the Quality of Life Scale for Children and Adolescents (QLSCA) [63]. This subscale consists of four items, three assessing parent-child interactions and one assessing youth’s perceived satisfaction of parental relationship. The three items assessing parent-child interactions are: (1) “How often do your parents spend time with you?” (2) “How often do you think your parents understand you?” and (3) “When coming across difficulties in life, how often are you willing to tell your parents?” (Answer options: 1 = “Never” and 4 = “Always”). The question assessing perceived satisfaction of parental relationship is: “How much are you satisfied with the relationship between you and your parents?” (1 = “not at all satisfactory” and 4 = “always satisfactory”). Cronbach alpha of this subscale was 0.77, and summed scores were computed for analysis such that higher scores indicated greater satisfaction of parent-child relationship.

#### Hyperactivity-Impulsivity

Hyperactivity-impulsivity was assessed using the Strengths and Difficulties Questionnaire (5 items) [64], [67]. We selected this measure because data from published studies indicate that SDQ scores were significantly correlated with depression, anxiety, and ADHD [68]. The five items assessing hyperactivity-impulsivity are: (1) “I am restless, and take a long time to calm down.” (2) “I am easily distracted, and I find it hard for me to concentrate.” (3) “I am constantly fidgeting or squirming.” (4) “I think before I do things.” and (5) “I finish the work I am doing. My attention is good.” The items were assessed using a 3-point scale with 0 = “Not agree”, 1 = “Not sure/don’t know”, and 2 = “Agree.” The Chinese version of the instrument was derived from the original SDQ (Goodman, 1997), and showed adequate reliability and validity among Chinese youth [64], [67]. Sum scores were computed after the two reversely stated items (4 and 5) were re-coded such that higher scores indicated greater hyperactivity and impulsivity.

#### Demographic variables

Age (in years), sex (male and female), school grade were included to describe the study sample.

### Statistical Analysis

Bivariate analysis (Student t-test and ANOVA for continuous variables and chi-square for categorical variables) was used to assess the relationship between the risk factors and levels of Internet addiction, as well as the interactions of parental relationship with age and hyperactivity-impulsivity. To assess the interaction of age with parental relationship, participants were categorized into younger adolescents and older adolescents with 14 years of age as the cutoff point; to assess the interaction of hyperactivity-impulsivity with parental relationship, this variable was dichotomized using the 90^th^ percentile as the cutoff point [69]. Results from the bivariate analysis were further verified using multiple linear regression method to include key covariates of age and gender. Type I error was set at *p*<0.05 level (two-tailed) in statistical analyses for hypothesis testing. Statistical analysis was conducted using the software SPSS version 18.0 (IBM SPSS Statistics).

## Results

### Characteristics of the Study Sample

Data for 1,101 participants (638 boys and 463 girls) with access to the Internet were included and they were accounted for 91.7% of the total sample. Results in Table 1 indicate that among the sample, approximately half were 13 years of age and younger with a mean age of 12.8 (SD = 1.2) years. The mean YIAT-C score was 36.0 (SD = 11.9) for the total sample, and boys scored significantly higher than girls (*t* = 5.1, *p*<0.001). There was no significant gender difference in the perceived parental relationship (*t = *0.5, *p = *0.623) and hyperactivity-impulsivity scores (*t* = −1.6, *p = *0.109).

**Table 1:** Characteristics of the Study Sample.

Characteristics	Male	Female	Total
**Sample size**, n (%)	638(57.9)	463 (42.1)	1101 (100.0)
**Age in years**			
13 and younger, n (%)	313 (55.4)	252 (44.6)	565 (100)
14 and older, n (%)	325(60.6)	211(39.4)	536(100)
Mean (SD)	13.8(1.2)	13.7(1.2)	13.8(1.2)
**School grade**, n (%)			
Grade 7	326(57.6)	240(42.4)	566(100)
Grade 8	129(58.4)	94(41.6)	221(100)
Grade 9	183(58.3)	131(41.7)	314(100)
YIAT-C score**			
Mean score (SD)	37.4(12.6)	33.9(10.7)	36.0(11.9)
**Parental relationship**			
Mean score (SD)	46.6(10.8)	46.3 (11.9)	46.5(11.3)
**Hyperactivity-Impulsivity**			
Mean score (SD)	3.6(2.1)	3.9(2.2)	3.7(2.1)

**Note:** **: p<0.01 from t-test.

### Prevalence of Addictive Internet Use

Table 2 summarizes the estimated prevalence rates of addictive Internet usage. Overall, 149 (13.5%, 95% CI: 11.5, 15.5%) respondents were classified as addicted Internet users. A chi-square test indicated that the prevalence rates were significantly higher for males than for females (16.5% vs. 9.5%, *p*<0.01) and significantly higher for older adolescents than for younger adolescents (15.7% vs. 11.5%, *p*<0.05). There was a significant increasing trend in the prevalence rate of addictive Internet use with school grades (*p*<0.05 from Cochran Armitage trend test).

**Table 2:** Prevalence of Addictive Internet Use among Adolescents with Access to Internet, 2009, Wuhan, China (N = 1101).

Category	Addictive Internet User YIAT-C score ≥50 n(%, 95% CI)	Non-Addictive Users YIAT-C score <50 n(%, 95% CI)	?^2^	*p*
**Total sample**	149(13.5%, 11.5–15.5%)	952(86.5%, 84.5–88.5%)		
**By Gender**			11.09	0.001
Male	105(16.5%, 13.6–19.4%)	533(83.5%, 80.6–86.4%)		
Female	44(9.5%, 6.8–12.2%)	419(90.5%, 87.8–93.2%)		
**By age group**			4.08	0.043
13 and younger	65(11.5%, 8.9–14.1%)	500(88.5%, 85.9–91.1%)		
14 and older	84(15.7%, 12.6–18.8%)	452(84.3%, 81.2–87.4%)		
**By school grade^a^**				
Grade 7	67(11.8%, 9.1–14.5%)	499(88.2%, 85.5–90.9%)	2.91	0.233
Grade 8	33(14.9%, 10.2–19.6%)	188(85.1%, 80.4–89.8%)		
Grade 9	49(15.6%, 11.6–19.6%)	265(84.4%, 80.4–88.4%)		

**Note**: **^a^** *Z* = 1.64, *p*<0.05 from Cochran-Armitage Trend Test.

### Association of Parental Relationship and Hyperactivity-Impulsivity with Internet Addiction

Results in Table 3 indicate that the addictive Internet users scored significantly lower on the parental relationship compared to the non-addictive users for the overall sample (*t* = 2.11, *p*<0.05), for female respondents (*t* = 2.56, *p* = 0.01), for younger respondents (*t* = 2.48, *p* = 0.01), and for respondents in grade nine (*t* = 2.00, *p*<0.05).

**Table 3:** Differences in Parental Relationship and Hyperactivity-Impulsivity between Addictive and Non-Addictive Internet Users, Middle School Students, 2009, Wuhan, China.

Group comparison	Parental relationship Mean (SD)	t (p value)	Hyperactivity-Impulsivity Mean (SD)	t (p value)
**Total sample (N = 1101)**				
Addictive users (n = 139)	44.58(11.71)	2.11(0.03)	5.21(1.97)	9.12(<0.01)
Non-addictive users (n = 934)	46.74(11.16)		3.51(2.05)	
**Gender**				
Males				
Addictive users (n = 98)	45.77(11.84)	0.84(0.40)	5.19(1.94)	8.33(<0.01)
Non-addictive users (n = 526)	46.76(10.55)		3.36(1.98)	
Females				
Addictive users (n = 43)	41.74(11.03)	2.56(0.01)	5.26(2.06)	4.55(<0.01)
Non-addictive users (n = 409)	46.71(11.91)		3.70(2.13)	
**Age group**				
≤13 years old				
Addictive users (n = 62)	43.05(12.03)	2.48(0.01)	5.42(1.81)	7.83(<0.01)
Non-addictive users (n = 495)	47.04(11.58)		3.31(2.02)	
≥14 years old				
Addictive users (n = 81)	45.68(11.43)	0.55(0.58)	5.04(2.09)	5.07(<0.01)
Non-addictive users (n = 439)	46.39(10.68)		3.74(2.07)	
**School Grade**				
Grade 7				
Addictive users (n = 62)	46.14 (11.82)	0.58(0.56)	5.31 (1.83)	7.02 (<0.01)
Non-addictive users (n = 495)	47.10 (11.6 2)		3.38 (2.06)	
Grade 8				
Addictive users (n = 32)	43.49 (13.55)	1.16(0.25)	5.25(2.17)	3.50(<0.01)
Non-addictive users (n = 184)	46. 08 (11.19)		3.84(2.10)	
Grade 9				
Addictive users (n = 48)	43.33(10.23)	2.01(<0.05)	5.05(2.03)	4.68(<0.01)
Non-addictive users (n = 256)	46.57(10.22)		3.52(2.00)	

**Note:** The number of addictive users and non-addictive users in each subgroup did not add up to the total sample because of missing data.

Compared to non-addictive users, addictive Internet users scored significantly higher on the hyperactivity-impulsivity item for the total sample and for all subgroups by gender, age and school grade. For example, among male respondents, the addicted users scored greater than non-addicted users on the hyperactivity-impulsivity items (5.19 vs. 3.36, *p*<0.01). Similar differences were also observed for students 13 years of age or younger and students in grade seven.

Results from multiple linear regression analysis in Table 4 indicated that parental relationship (b = −0.07, p<0.05) and hyperactivity-impulsivity (b = 1.95, p<0.01) were significant predictors of YIAT-C scores after controlling for age, and gender when the interaction was not considered (Model I).

**Table 4:** Factors Associated with Addictive Internet Use among Adolescents in Wuhan, China.

Variables	Model I (with no interaction) Coefficient (P)	Model II (with interaction) Coefficient (P)
Age	1.02 (0.001)	1.76(<0.001)
Gender	−3.55(<0.001)	−3.42(<0.001)
Parental relationship	−0.07(0.017)	−0.22(0.548)
Interaction with age	/	−0.02(<0.001)
Hyperactivity - Impulsivity	1.95(<0.001)	0.12(0.286)
Interaction with parental relationship	/	0.04(<0.001)
Model fit, F(df), p value	Multiple R = 0.41, R^2^ = 0.172, Adjusted R^2^ = 0.169, F(4) = 53.61, *p*<0.001	Multiple R = 0.42 R^2^ = 0.174, Adjusted R^2^ = 0.171 F(4) = 54.64, *p*<0.001

**Note**: here age is a continuous variable. However, age variable in the interaction figure is segment into two.

### Modification Effect of Adolescents’ Age on Parental Relationship

**Figure 1:**
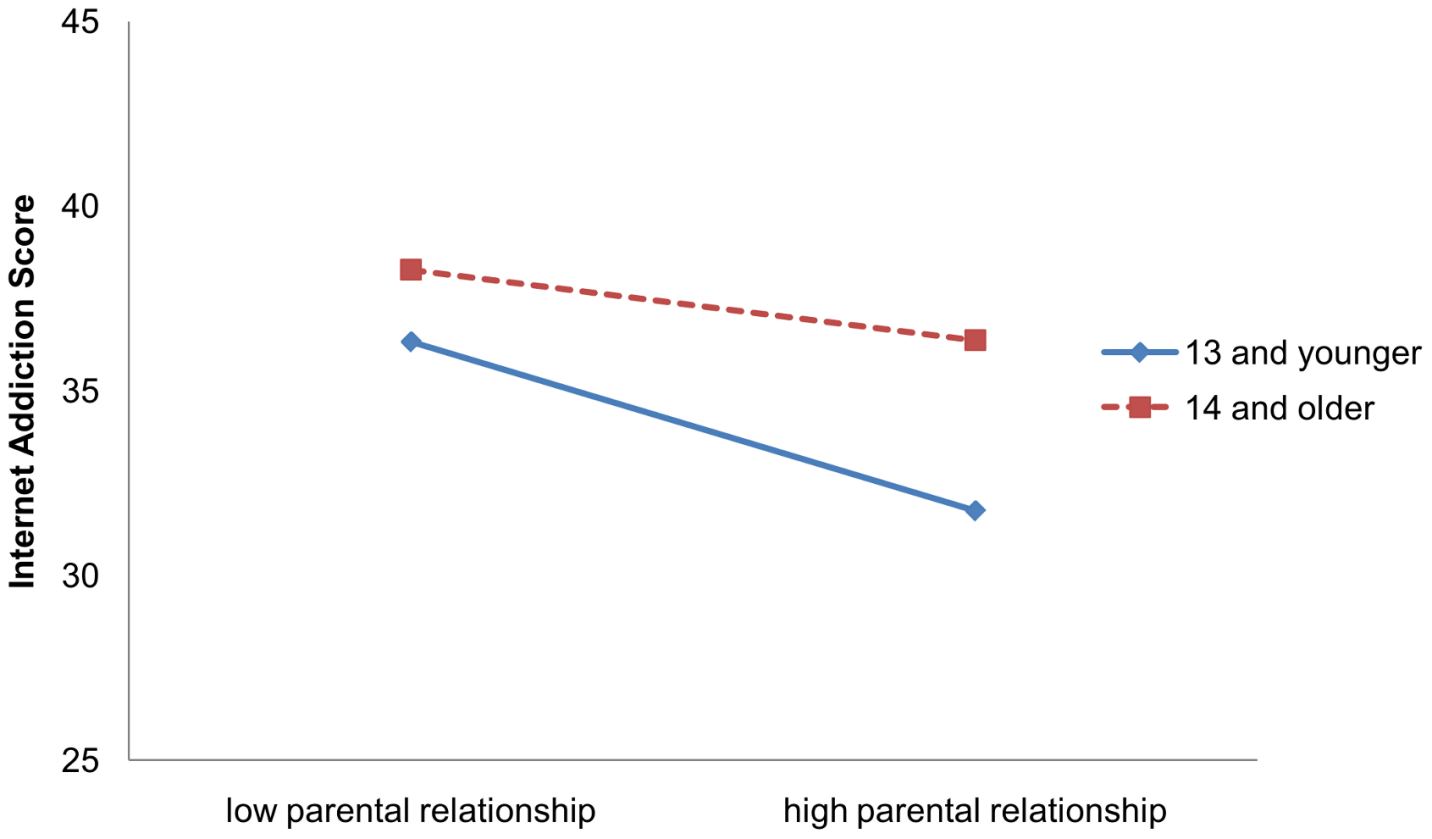
Interaction of age with parental relationship on Internet Addiction. In figure 1, the continuous blue line is for 13 and younger, and the dashed red line is for 14 and older.

**Figure 2:**
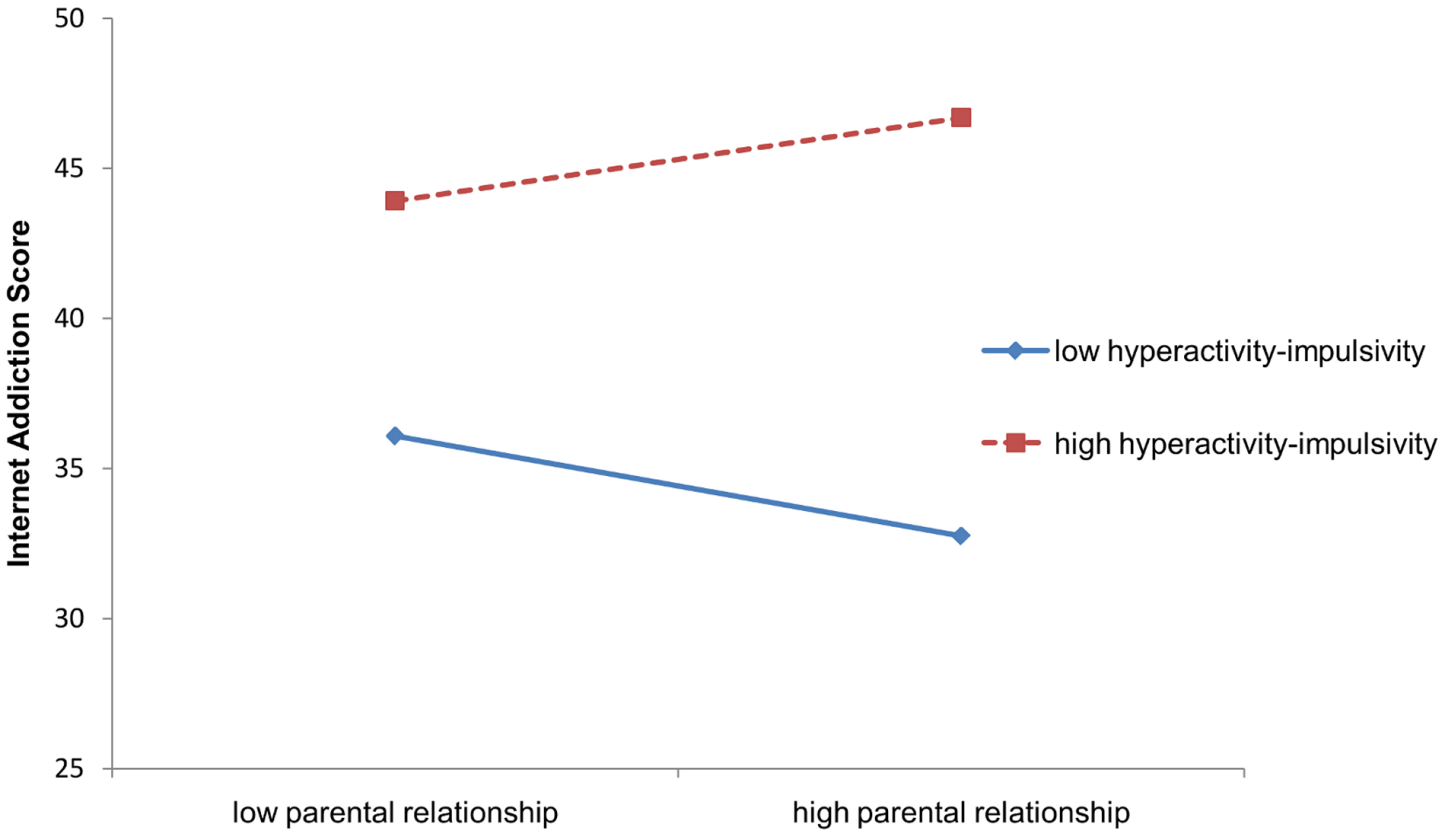
Interaction of hyperactivity-impulsivity with parental relationship on Internet Addiction. In figure 2, the continuous blue line is for low hyperactivity-impulsivity, and the dashed red line is for high hyperactivity-impulsivity.

Model II results in Table 4 indicate that parental relationship was negatively interacted with age (b = −0.02, p<0.01) and positively interacted with hyperactivity-impulsivity (b = 0.04, p<0.01) after controlling for age, gender and the main effect of these two variables. Figure 1 and Figure 2 present the two interactive effects.

## Discussion and Conclusions

Addictive Internet use is a global public health challenge and this issue is particularly urgent in China. There is an explosive growing number of netizens in China, currently totaling more than 500 million. In this study, we reported the results from a survey study we conducted regarding addictive Internet use and influential factors in Wuhan, China. Findings of this study add new data for us to understand the role of parental relationship and its interaction with age and hyperactivity-impulsivity in affecting the likelihood of adolescent Internet addiction.

### High Prevalence of Internet Addiction

Findings from our analysis indicated that 13.5% of Chinese middle school students with access to Internet were addicted to the Internet. This rate was higher than those rates recently reported among high school students (6.4%) and college students (12.2%) in China using the same YIAT-C measure [8], [12], [70]. The findings of our studies imply that hundreds of millions of young Chinese adolescents are now addicted to Internet. The resultant health and social consequences would be enormous if no immediate and rigorous intervention measures are taken to curb this epidemic. The findings from our analysis indicated that adolescents with male gender and younger ages are at disproportionately high risk of developing Internet addiction. Therefore, they consist of a priority subpopulation for an Internet addiction prevention intervention.

### Significance Role of Parental Relationship in Adolescent Internet Addiction

Findings of this study support our hypothesis that poor parental relationship was associated with increased likelihood of addictive Internet use among Chinese adolescents. Compared to non-addicted Internet users, addicted users scored significantly lower on the Parental Relationship Scale and there was a significant negative association between the Parental Relationship Scale scores and YIAT-C scores. These findings imply that the risk for Internet addiction will be greater for students who do not often spend time with their parents, do not feel that their parents understand them, and do not disclose problems to their parents. Findings of our study strengthened the conclusion from previous research on the risk of poor parental relationship on addictive Internet usage among adolescents [26]–[28], [34] and expanded the data on parent-child interaction and communication and health risk behaviors in general.

### Interaction of Parental Relationship with Other Influential Factors

A unique finding from our study is that the association between parental relationship and addictive Internet use is not homogeneous but varies by several key intrapersonal factors, including age, gender and hyperactivity-impulsivity traits. Findings from our analyses, including simple comparative analysis, ANOVA and multiple linear regression indicate that although adolescents with a better parental relationship were less likely to become addictive internet user, the association differed by age and levels of hyperactivity-impulsivity. The negative association between parental relationship and Internet addiction was stronger for students 13 years of age or younger relative to students aged 14 and older. Better parental relationship was associated with increased risk of Internet addiction among students with higher levels of hyperactivity-impulsivity than students with lower levels.

### Implications for Prevention Interventions

The findings of the negative association between parental relationship and Internet addiction suggest the potential role of promoting good parental relationship in curbing the epidemic addictive Internet use among Chinese adolescents. An effective prevention intervention curriculum should incorporate contents to promote parent-child communication, encourage parents to spend time with their teenage children, and educate them to understand the needs of their adolescent children, including Internet usage. Reported studies, including randomized controlled trials suggest the potentials to reduce addictive internet use through behavioral intervention and counseling. [71]–[73].

In addition to targeting adolescents in general, extra attention should be paid to several high-risk subgroups while devising intervention to enhance parenting skills for Internet addiction prevention. Findings of this study imply that Chinese male youth in their later-teens with hyperactivity-impulsivity tendency may not be very responsive to improvement in parental relationship with regard to prevention of Internet addiction. In this case, additional actions may be needed to deal with age-related developmental issues and issues related to hyperactivity-impulsivity.

There are several limitations to this study. First, data used for this study is cross-sectional in nature. Therefore, causal impact of parental relationship on Internet addiction is not warranted without longitudinal verification. Second, Internet addiction was not determined by certified clinicians. YIAT reflects mainly the DSM-IV’ criteria of addiction, the results may differ if other criteria such as the International Statistical Classification of Diseases and Related Health Problems (ICD) were used. Third, we used self-reported data from adolescents’ report only. Information bias could not be assessed without information from other sources, such as reports from parents and other informants (e.g., care givers, teachers, and clinicians). Lastly, data was collected from one city in China. Although diversity was considered in school selection within the city, caution is advised if findings from this study are to be generalized to students in other parts of China.

Despite the limitations, findings of this study provided data to understand the significance of parental relationship as well as its interaction with intrapersonal factors of age and hyperactivity-impulsivity in predicting addictive Internet use. Such data are important for public health decision makers and health behavior scientists to plan and devise intervention programs to control the Internet addiction epidemic in China. To care adolescents who are already addicted to Internet, additional studies are needed to measure the dependence and other influential factors using assessment tools relevant for use at clinic setting.
